# Artificial intelligence versus human expertise: reliability of ChatGPT and the London atlas for dental age estimation using panoramic radiographs

**DOI:** 10.1186/s12903-025-07360-w

**Published:** 2025-11-28

**Authors:** Ramazan Berkay Peker

**Affiliations:** Department of Dentomaxillofacial Radiology, Trakya University Faculty of Dentistry, Balkan Campus, Edirne, 22030 Turkey

**Keywords:** ChatGPT, London atlas, Panoramic radiography, Dental age estimation, Artificial intelligence, Reliability

## Abstract

**Background:**

This study evaluated the performance of ChatGPT, a multimodal large language model (LLM), in estimating dental age from panoramic radiographs (PRs) and compared its accuracy and reproducibility with those of the London Atlas (LA) method.

**Methods:**

PRs of 620 healthy children aged 6 through 13 years were retrospectively analyzed. An experienced dentomaxillofacial radiologist estimated dental age using the LA, and the ChatGPT-4o model analyzed the same anonymized images to generate automated age predictions. Both methods were repeated after two weeks to assess intra-observer reliability. Predictive accuracy and agreement with chronological age (CA) were evaluated using mean absolute error (MAE), root mean squared error (RMSE), intraclass correlation coefficients (ICC), and Bland–Altman analyses. Statistical significance was set at *p* < .05.

**Results:**

ChatGPT’s predictions differed significantly from chronological age (CA), tending to overestimate age in younger children and underestimate age in older children. Compared with the LA, ChatGPT exhibited higher MAE and RMSE values, indicating lower predictive accuracy and greater variability. Error magnitudes were greatest in the 6-, 12-, and 13-year-old groups and lowest in the 8-year-old group, whereas the LA showed lower and more stable errors across ages. The LA demonstrated fewer discrepancies and excellent reproducibility (ICC = 0.960) as compared with the moderate agreement of ChatGPT (ICC = 0.703). Overall, the LA provided estimates closer to CA, whereas ChatGPT exhibited greater variability.

**Conclusions:**

ChatGPT shows promise for complex decision-making tasks such as dental age estimation; however, its current accuracy, reproducibility, and output stability remain inferior to established methods such as the LA. The inconsistent predictions observed across repeated evaluations highlight a critical limitation regarding its reliability for clinical and forensic applications. Therefore, ChatGPT-based estimations should be interpreted with caution until future versions achieve more consistent and reproducible performance through population-specific training, model optimization, and multicenter validation.

## Background

Accurate estimation of an individual’s chronological age (CA) is of paramount importance across numerous clinical and legal domains, including forensic medicine, criminal law, immigration assessment, and orthodontic treatment planning [1–3]. Consequently, age estimation methods must be accurate, reproducible, and applicable to the target population. Among the biological indicators used for age assessment, dental development is recognized as a particularly reliable marker in children and adolescents because of its strong genetic control and lesser susceptibility to environmental, systemic, and hormonal influences [4, 5]. Teeth remain well preserved even after burial and exhibit less biological variation than skeletal structures, making dental development one of the more reliable biological indicators for age estimation, particularly during childhood and adolescence [6].

Currently, dental age estimation is conducted using morphological, scoring, metric, and atlas-based approaches. Among these, the London Atlas (LA) is widely preferred owing to its ease of application, inclusion of all jaw quadrants, and rapidly generated results [7]. This evidence-based reference enables age estimation based on dental development and alveolar eruption from the 28th week of gestation to 23 years of age [6]. Moreover, unlike earlier atlases, the LA integrates visual and radiographic data across the full dentition period, offering high repeatability and practical applicability.

Nevertheless, the LA has been faulted for being sex insensitive, showing limited sensitivity to inter-population variation, and occasionally producing systematic bias in age estimation [8]. Such discrepancies, particularly near the critical legal threshold of 12 years for criminal responsibility in many countries, can have significant forensic and legal consequences [9]. Another key limitation of classical methods is observer dependency: Subjective interpretation of dental development stages, differences in observer experience, inadequate calibration, and ambiguities in stage definitions all contribute to high inter- and intra-observer variability, thereby reducing methodological reliability [10–14].

These limitations have inspired increasing interest in the application of artificial intelligence (AI)-based automated systems for age estimation. Deep learning models, particularly convolutional neural networks (CNNs), can with high accuracy automatically classify dental development stages from panoramic radiographs (PRs). Two-stage deep learning approaches have demonstrated clinically acceptable accuracy and strong concordance with manual expert assessments [15].

More recently, large language models (LLMs) have been integrated into visual data analysis workflows. The GPT-4 V and GPT-4o versions of ChatGPT (OpenAI, San Francisco, CA, USA) are multimodal systems capable of performing not only text-based reasoning but also image-based assessments, and exploration of their potential in the diagnostic interpretation of dental radiographs has yielded promising results. For instance, GPT-4 V, GPT-4o, and a customized GPT-4 V model were evaluated for supernumerary tooth detection in periapical radiographs, and all achieved accuracies exceeding 60%, representing clinically acceptable performance [16]. These findings indicate that even ChatGPT models trained on limited visual data can make clinically relevant decisions directly from radiographic images.

To the best of our knowledge, however, no study has evaluated the performance of ChatGPT or similar LLMs in multifactorial decision-making tasks such as age estimation. This research gap underscores the need for systematic investigation of these models. The present study performed ChatGPT-based age estimation using PRs of pediatric patients in the Thrace region of Turkey. The same radiographs were also evaluated using the human-observer–based LA method, and the two approaches were comparatively analyzed. Furthermore, ChatGPT’s intra-observer reliability in repeated assessments of identical radiographs was examined and compared with human observer performance. The aim of this study was to compare the accuracy, reliability, and consistency of the LA– and ChatGPT-based age estimation methods using PRs of children from the Thrace region of Türkiye and to explore the potential of ChatGPT in dental age estimation.

## Methods

### Study design and ethical approval

This retrospective observational study involved no interventions. Data were obtained from digital PRs archived in the Department of Dentomaxillofacial Radiology, Faculty of Dentistry, Trakya University. All radiographs were anonymized by removing patient identifiers, such as names and protocol numbers. The study protocol was approved by the Non-Interventional Scientific Research Ethics Committee of Trakya University Faculty of Medicine (No. TÜTF-GOBEAK 2025/376) and was conducted in accordance with the principles of the Declaration of Helsinki and Turkey’s Personal Data Protection Law.

### Study population

The study included 620 children who underwent PR for diagnostic purposes between 1 January 2015 and 31 December 2024. The required sample size was calculated using G*Power software (version 3.1.9.7) (Heinrich Heine University, Düsseldorf, Germany). Based on data from a relevant reference study, a minimum of 394 participants was required to achieve a 95% confidence level (1 − α), 95% statistical power (1 − β), and an effect size of Cohen’s *dz* = 0.182 [9]. In the present study, 620 radiographs were evaluated, yielding a post hoc statistical power of 99.49%.

Cases were included if they were aged 6 through 13 years at the time of imaging, had sex information clearly documented in the archive records, and presented diagnostic-quality PRs with clearly visible permanent tooth buds. Individuals were excluded who had a history of systemic diseases, genetic syndromes, maxillofacial trauma or surgery, radiotherapy or chemotherapy, developmental disorders or anomalies, or missing or supernumerary teeth. Additional exclusion criteria comprised multiple permanent tooth extractions, anatomical variations, pathological formations, tumors or cystic lesions visible on PRs, severe carious destruction, trauma or artefacts that could compromise age estimation, and dental anomalies, such as transposition, root resorption, ankylosis, or arrested tooth development. Radiographs of insufficient image quality for reliable analysis were also excluded.

### Participant characteristics

A total of 620 children were included, comprising 310 boys and 310 girls. Their ages ranged from 6 to 13 years, with a mean age of 10.12 ± 2.24 years and a median age of 10.29 years. Table 1 shows age and sex distributions.

**Table 1:** Age- and sex-specific distribution of pediatric participants (*n*, %)

Age (years)	Boys *n*, (%)	Girls *n*, (%)	Total *n*, (%)
6.00–6.99	33 (10.6)	30 (9.7)	63 (10.2)
7.00–7.99	40 (12.9)	47 (15.2)	87 (14)
8.00–8.99	33 (10.6)	29 (9.4)	62 (10)
9.00–9.99	42 (13.5)	31 (10)	73 (11.8)
10.00–10.99	39 (12.6)	49 (15.8)	88 (14.2)
11.00–11.99	43 (13.9)	44 (14.2)	87 (14)
12.00–12.99	44 (14.2)	40 (12.9)	84 (13.5)
13.00–13.99	36 (11.6)	40 (12.9)	76 (12.3)

Age- and sex-specific distribution of the study population, expressed as numbers (*n*) and percentages (%)

### Image acquisition and data preparation

All PRs were acquired by the same radiology technician with the Frankfort horizontal plane aligned parallel to the floor and the midsagittal plane correctly positioned. Images were obtained using a Vatech PaX-Flex digital panoramic system (Vatech, Seoul, South Korea) with exposure parameters of 50–90 kV and 4–10 mA and an exposure time of 10.1 s. To preserve image quality for digital analyses, radiographs were converted to portable network graphics (PNG) format and irreversibly anonymized before analysis. All images were handled exclusively by the primary researcher.

Prior to uploading into ChatGPT, only these fully de-identified images were used. All potentially identifiable information—including patient names, accession numbers, facial outlines, or embedded metadata—had been permanently removed. The data transfer was performed strictly within the scope of the approved ethics protocol and in accordance with institutional and national data-protection regulations.

### Chronological age calculation

CA was calculated as the difference between the date of PR and the date of birth, expressed in years to two decimal places. The calculation was performed in Microsoft Excel 2016 (Microsoft, Redmond, WA, USA) using the following formula:

[(Date of radiograph acquisition) − (Date of birth)] ÷ 365.25.

### Age estimation methods

#### London Atlas–based method

All PRs were evaluated by a single oral and maxillofacial radiologist (R.B.P.) using the LA method. Dental development stages were assessed according to the instructions of AlQahtani et al. [6]. Each image was compared with the reference figures provided in the LA software (https://www.qmul.ac.uk/dentistry/atlas/software-app-full-width-/), and the age corresponding to the closest developmental stage was recorded as the dental age [17].

#### ChatGPT-based method

The same anonymized images were analyzed using ChatGPT-4o, an LLM with multimodal capabilities. Each radiograph, along with the patient’s sex information, was uploaded to the model. The model was instructed with the following English prompt: “Evaluate the dental development stages in the provided PR, considering that the patient is female, and estimate the patient’s CA with maximum precision as a single value. Fractional age predictions are allowed, but ranges are not accepted.” This instruction directed the model to evaluate dental development and generate a single CA prediction with maximum precision.

No additional or customized prompts were used during the analyses. Apart from the standardized instruction described above, ChatGPT received no external guidance, and it was not directed to refer to the LA or any other atlas-based resource. All estimations were generated autonomously based solely on the provided PRs and the patient’s sex information.

As ChatGPT-4o operates through a proprietary multimodal interface rather than the OpenAI Application Programming Interface (API), sampling parameters such as temperature, top-p, and seed could not be manually defined or fixed by the user. Therefore, the outputs may inherently include a degree of stochastic variation. This limitation was considered in interpreting the reproducibility results.

### Reproducibility analysis

All analyses were repeated two weeks later using both the LA and ChatGPT methods to assess intra-observer reliability (test-retest consistency) for both human and AI-based evaluations.

### Statistical analysis

Statistical analyses were performed using IBM SPSS Statistics version 23 (IBM Corp., Chicago, IL, USA) and R version 4.4.1 (R Foundation for Statistical Computing, Vienna, Austria). Data normality was assessed using the Kolmogorov–Smirnov and Shapiro–Wilk tests. For normally distributed data, paired-sample *t*-tests were applied; for non-normal distributions, the Wilcoxon signed-rank test was used. Agreement between measurements was quantified using the intraclass correlation coefficient (ICC) and further evaluated using the Bland–Altman method to assess the mean bias and limits of agreement between paired measurements. Prediction accuracy was additionally quantified using the mean absolute error (MAE) and root mean squared error (RMSE), which measure the average and squared magnitudes of the prediction errors, respectively. Continuous variables were expressed as mean ± standard deviation (SD) and median (minimum–maximum), whereas categorical variables were presented as frequency and percentage (%). A *p*-value of < 0.05 was considered statistically significant.

## Results

### Reproducibility of ChatGPT age estimations

**Fig. 1:**
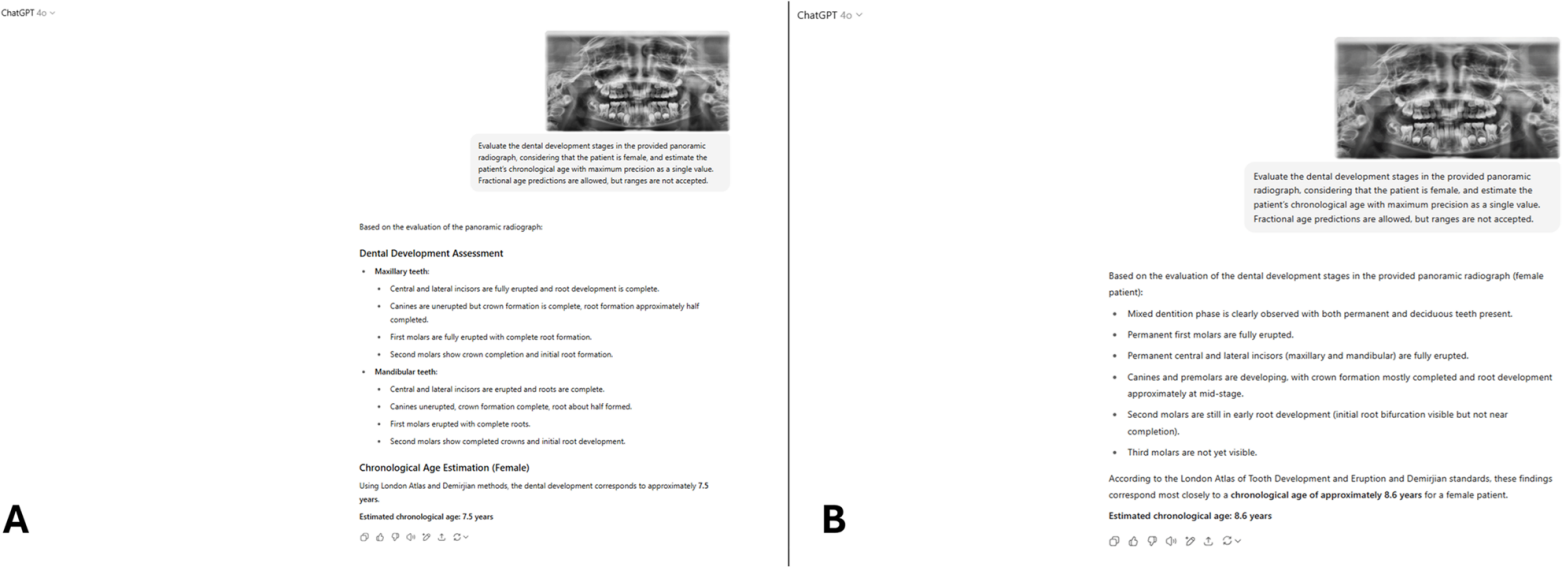
ChatGPT’s two independent age estimations of the same panoramic radiograph. **A** First estimation performed by ChatGPT. **B** Second estimation performed two weeks later from the same panoramic radiograph. Both estimations were generated autonomously without any reference to the London Atlas

When ChatGPT’s first- and second-round age estimations were compared, the median predicted age in boys increased from 9.1 to 9.5 years (*p <*.001). In girls, the median prediction increased from 9.6 to 9.8 years (*p =*.017), and in the overall sample it increased from 9.3 to 9.6 years (*p <*.001). Agreement between the repeated ChatGPT estimations was moderate (boys: ICC = 0.712, 95% Confidence Interval (CI): 0.653–0.763; girls: ICC = 0.696, 95% CI: 0.633–0.749; overall: ICC = 0.703, 95% CI: 0.660–0.740; all *p <*.001) (Table 2). Figure 1 shows two ChatGPT age estimations of the same PR performed at different time points, which produced discrepant results.

**Table 2:** Reproducibility of age estimation using ChatGPT and the London Atlas with error and reliability metrics

		1 st Estimation			2nd Estimation			Test Statistic	*pₓ Value*	ICC (95% CI)	*pγ Value*
		Median (Min–Max)	MAE	RMSE	Median (Min–Max)	MAE	RMSE				
Boys	ChatGPT	9.10 (6.10–16.00)	1.47	1.86	9.50 (6.50–15.20)	1.37	1.77	−6.103	< 0.001	0.712 (0.653–0.763)	< 0.001
	London Atlas	10.50 (6.50–15.50)	0.77	1.04	10.50 (6.50–15.50)	0.79	1.06	−1.896	0.058	0.960 (0.950–0.968)	< 0.001
Girls	ChatGPT	9.60 (5.80–14.10)	1.47	1.82	9.80 (6.20–15.10)	1.45	1.82	−2.385	0.017	0.696 (0.633–0.749)	< 0.001
	London Atlas	10.50 (6.50–15.50)	0.95	1.41	10.50 (6.50–15.50)	0.91	1.36	−1.385	0.166	0.960 (0.950–0.968)	< 0.001
Overall	ChatGPT	9.30 (5.80–16.00)	1.47	1.84	9.60 (6.20–15.20)	1.41	1.80	−6.049	< 0.001	0.703 (0.660–0.740)	< 0.001
	London Atlas	10.50 (6.50–15.50)	0.86	1.24	10.50 (6.50–15.50)	0.85	1.22	−2.316	0.021	0.960 (0.953–0.966)	< 0.001

Median values with minimum and maximum ranges are presented

*MAE* mean absolute error, *RMSE* root mean square error, *x* Wilcoxon signed-rank test, *y* intraclass correlation coefficient (ICC) with 95% confidence intervals (*CI*)

The MAE and RMSE for the first and second ChatGPT estimations were 1.47 and 1.86 years in boys, 1.47 and 1.82 years in girls, and 1.47 and 1.84 years overall for the first estimations; and 1.37 and 1.77 years in boys, 1.45 and 1.82 years in girls, and 1.41 and 1.80 years overall for the second estimations, respectively (Table 2).

**Fig. 2:**
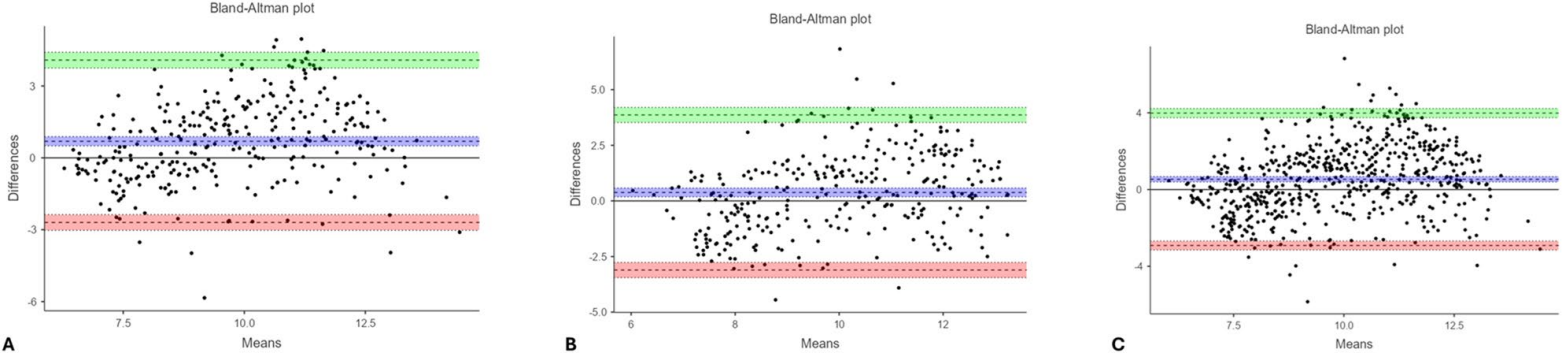
Bland–Altman plots illustrating the agreement between chronological age and ChatGPT first measurement values: (**A**) males, (**B**) females, and (**C**) the overall sample (regardless of sex). In each plot, the x-axis represents the mean of the two measurements, and the y-axis represents their difference. The green area denotes the upper limit of agreement, the red area indicates the lower limit, and the blue area corresponds to the 95% confidence interval of the mean difference. Each dot represents an individual observation; values falling between the green and red boundaries indicate acceptable agreement between chronological age and ChatGPT-based estimations

**Fig. 3:**
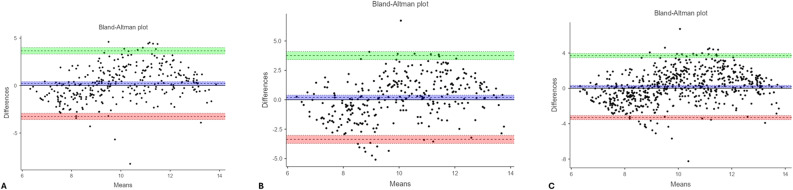
Bland–Altman plots illustrating the agreement between chronological age and ChatGPT second measurement values: (**A**) males, (**B**) females, and (**C**) the overall sample (regardless of sex). In each plot, the x-axis represents the mean of the two measurements, while the y-axis represents their difference. The green area denotes the upper limit of agreement, the red area indicates the lower limit, and the blue area corresponds to the 95% confidence interval of the mean difference. Each dot represents an individual observation; values lying between the green and red boundaries indicate acceptable agreement between chronological age and ChatGPT-based estimations

Bland–Altman analysis demonstrated moderate agreement in both rounds (Figs. 2 and 3), with limits of agreement (LOAs) between − 2.913 and + 3.984 years for the overall sample (Table 3).

**Table 3:** Bland–Altman analysis for agreement between chronological age and estimated ages

Measurements	Bland–Altman parameters	Boys	Girls	Overall
Chronological age – ChatGPT 1 st estimation	Lower LOA	−2.697 (−3.028 – −2.367)	−3.106 (−3.446 – −2.766)	−2.913 (−3.150 – −2.676)
	Upper LOA	4.082 (3.751–4.412)	3.864 (3.524–4.204)	3.984 (3.747–4.221)
Chronological age – ChatGPT 2nd estimation	Lower LOA	−3.250 (−3.587 – −2.913)	−3.363 (−3.710 – −3.016)	−3.304 (−3.545 – −3.063)
	Upper LOA	3.660 (3.323–3.997)	3.753 (3.406–4.101)	3.704 (3.463–3.945)
Chronological age – London 1 st estimation	Lower LOA	−2.234 (−2.426 – −2.041)	−2.994 (−3.259–−2.730)	−2.641 (−2.805 – −2.478)
	Upper LOA	1.708 (1.516–1.900)	2.436 (2.171–2.700)	2.099 (1.936–2.262)
Chronological age – London 2nd estimation	Lower LOA	−2.312 (−2.505 – −2.119)	−2.929 (−3.182 – −2.676)	−2.639 (−2.798 – −2.481)
	Upper LOA	1.644 (1.451–1.837)	2.267 (2.013–2.520)	1.975 (1.816–2.133)

Limits of agreement (LOAs) are presented with 95% confidence intervals

### Reproducibility of London Atlas age estimations

The LA method yielded identical median values of 10.5 years for both boys and girls, with no statistically significant differences between the first- and second-round estimations (boys: *p =*.058; girls: *p =*.166). Although a minor difference was significant in the overall sample (*p =*.021), the unchanged median values suggest that this difference is attributable only to slight shifts in rank averages and lacks clinical relevance. The LA demonstrated excellent reproducibility (boys: ICC = 0.960, 95% CI: 0.950–0.968; girls: ICC = 0.960, 95% CI: 0.950–0.968; overall: ICC = 0.960, 95% CI: 0.953–0.966; all *p <*.001) (Table 2).

The MAE and RMSE between the first and second LA estimations were 0.77 and 1.04 years in boys, 0.95 and 1.41 years in girls, and 0.86 and 1.24 years overall for the first estimations; and 0.79 and 1.06 years in boys, 0.91 and 1.36 years in girls, and 0.85 and 1.22 years overall for the second estimations, respectively (Table 2).

**Fig. 4:**
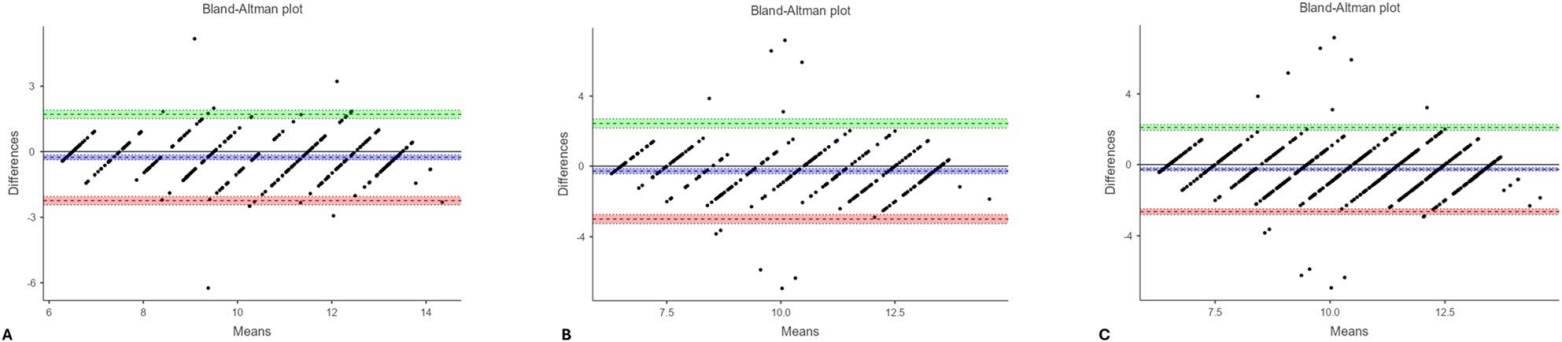
Bland–Altman plots illustrating the agreement between chronological age and London Atlas first measurement values: (**A**) males, (**B**) females, and (**C**) the overall sample (regardless of sex). In each plot, the x-axis represents the mean of the two measurements, and the y-axis represents their difference. The green area denotes the upper limit of agreement, the red area indicates the lower limit, and the blue area corresponds to the 95% confidence interval of the mean difference. Each dot represents an individual observation; values located between the green and red boundaries indicate acceptable agreement between chronological age and London Atlas–based estimations

**Fig. 5:**
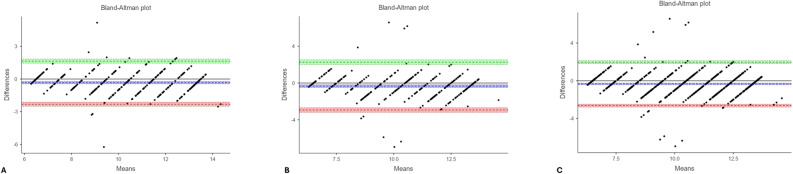
Bland–Altman plots illustrating the agreement between chronological age and London Atlas second measurement values: (**A**) males, (**B**) females, and (**C**) the overall sample (regardless of sex). In each plot, the x-axis represents the mean of the two measurements, and the y-axis represents their difference. The green area denotes the upper limit of agreement, the red area indicates the lower limit, and the blue area corresponds to the 95% confidence interval of the mean difference. Each dot represents an individual observation; values lying between the green and red boundaries indicate acceptable agreement between chronological age and London Atlas–based estimations

Bland–Altman analysis showed excellent reproducibility, with narrow LOA between − 2.639 and + 1.975 years for the overall sample (Table 3; Figs. 4 and 5).

### Comparison of ChatGPT and London Atlas

**Fig. 6:**
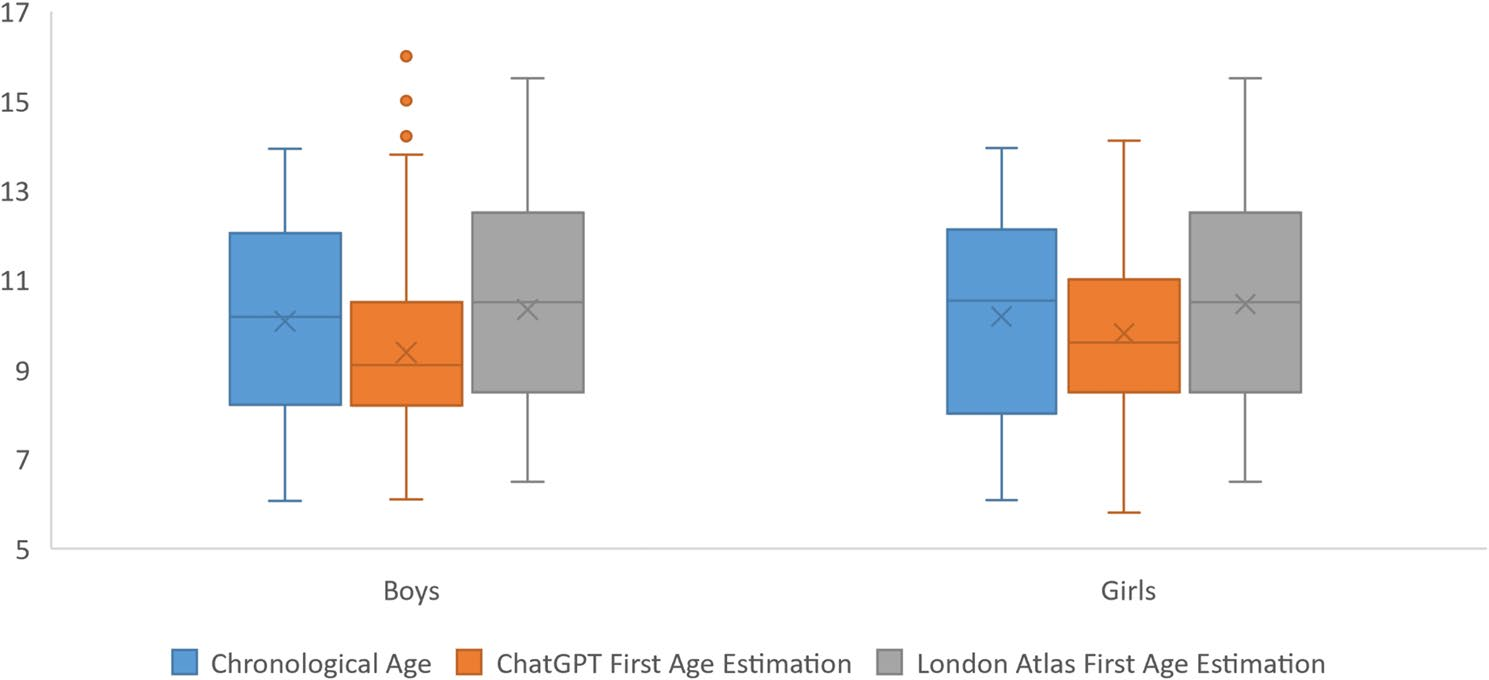
Comparison of chronological age in boys and girls with first age estimations by ChatGPT and London Atlas

In both rounds, LA estimations were significantly higher than ChatGPT results. Among boys, median ages were 9.1 years for ChatGPT and 10.5 years for the LA in the first-round estimations (*p <*.001; ICC = 0.658, 95% CI: 0.590–0.717) and 9.5 years and 10.5 years, respectively, in the second-round estimations (*p <*.001; ICC = 0.649, 95% CI: 0.579–0.709). Among girls, median ages were 9.6 years for ChatGPT and 10.5 years for the LA in the first-round estimations (*p <*.001; ICC = 0.691, 95% CI: 0.628–0.745) and 9.8 years and 10.5 years, respectively, in the second-round estimations (*p <*.001; ICC = 0.650, 95% CI: 0.581–0.710). In the overall sample, median ages estimated by ChatGPT were 9.3 years in the first round and 9.6 years in the second, compared with 10.5 years for the LA in both rounds (*p <*.001; ICC = 0.674 and 0.650, 95% CI: 0.628–0.714 and 0.602–0.693, respectively) (Table 4; Figs. 5 and 6).

**Table 4:** Comparison of ChatGPT- and London Atlas–based age estimations by sex and estimation round with error and reliability metrics

		ChatGPT			London Atlas			Test Statistic	*p*ₓ Value	ICC (95% CI)	*p*_γ_ Value
		Median (Min–Max)	MAE	RMSE	Median (Min–Max)	MAE	RMSE				
Boys	1 st estimation	9.10 (6.10–16.00)	1.47	1.86	10.50 (6.50–15.50)	0.77	1.04	−8.760	< 0.001	0.658 (0.590–0.717)	< 0.001
	2nd estimation	9.50 (6.50–15.20)	1.37	1.77	10.50 (6.50–15.50)	0.79	1.06	−5.494	< 0.001	0.649 (0.579–0.709)	< 0.001
Girls	1 st estimation	9.60 (5.80–14.10)	1.47	1.82	10.50 (6.50–15.50)	0.95	1.41	−6.683	< 0.001	0.691 (0.628–0.745)	< 0.001
	2nd estimation	9.80 (6.20–15.10)	1.45	1.82	10.50 (6.50–15.50)	0.91	1.36	−5.598	< 0.001	0.650 (0.581–0.710)	< 0.001
Overall	1 st estimation	9.30 (5.80–16.00)	1.47	1.84	10.50 (6.50–15.50)	0.86	1.24	−10.990	< 0.001	0.674 (0.628–0.714)	< 0.001
	2nd estimation	9.60 (6.20–15.20)	1.41	1.80	10.50 (6.50–15.50)	0.85	1.22	−7.869	< 0.001	0.650 (0.602–0.693)	< 0.001

Median values with minimum and maximum ranges are presented

*MAE* mean absolute error, *RMSE* root mean square error, *x* Wilcoxon signed-rank test, *y* intraclass correlation coefficient (*ICC*) with 95% confidence intervals (*CI*)

Error-based comparisons indicated that ChatGPT consistently produced higher MAE and RMSE values than the LA in both rounds. For boys, MAE and RMSE ranged from 1.47 to 1.37 and 1.86–1.77 years for ChatGPT, compared with 0.77–0.79 and 1.04–1.06 years for the LA. For girls, the corresponding values were 1.47–1.45 and 1.82 years for ChatGPT, versus 0.95–0.91 and 1.41–1.36 years for the London Atlas. In the overall sample, MAE and RMSE were 1.47–1.41 and 1.84–1.80 years for ChatGPT, and 0.86–0.85 and 1.24–1.22 years for the London Atlas (Table 4).

Bland–Altman plots illustrated wider limits of agreement for ChatGPT compared with the LA, further supporting this finding (Figs. 2, 3, 4 and 5).

### ChatGPT versus chronological age

ChatGPT’s first-round estimations were significantly lower than CA in boys (median: 9.1 years vs. 10.17 years; *p <*.001; ICC = 0.632), in girls (median: 9.6 years vs. 10.53 years; *p =*.001; ICC = 0.602), and in the overall sample (median: 9.3 years vs. 10.29 years; *p <*.001; ICC = 0.616) (Table 5; Fig. 6).

**Table 5:** Comparison of chronological age and ChatGPT-based age estimation with error and reliability metrics

		Mean ± SD	Median (Min–Max)	MAE	RMSE	Test Statistic	pₓ Value	ICC (95% CI)	*p*_γ_ Value
Boys	Chronological age	10.07 ± 2.24	10.17 (6.06–13.92)	1.47	1.86	−6.587	< 0.001	0.632 (0.561–0.695)	< 0.001
	ChatGPT 1 st estimation	9.38 ± 1.77	9.10 (6.10–16.00)						
	Chronological age	10.07 ± 2.24	10.17 (6.06–13.92)	1.37	1.77	−1.927	0.054	0.612 (0.538–0.677)	< 0.001
	ChatGPT 2nd estimation	9.87 ± 1.73	9.50 (6.50–15.20)						
Girls	Chronological age	10.18 ± 2.24	10.53 (6.08–13.95)	1.47	1.82	−3.369	0.001	0.602 (0.526–0.668)	< 0.001
	ChatGPT 1 st estimation	9.80 ± 1.70	9.60 (5.80–14.10)						
	Chronological age	10.18 ± 2.24	10.53 (6.08–13.95)	1.45	1.82	−2.126	0.034	0.583 (0.504–0.652)	< 0.001
	ChatGPT 2nd estimation	9.98 ± 1.69	9.80 (6.20–15.10)						
Overall	Chronological age	10.12 ± 2.24	10.29 (6.06–13.95)	1.47	1.84	−7.058	< 0.001	0.616 (0.565–0.663)	< 0.001
	ChatGPT 1 st estimation	9.59 ± 1.75	9.30 (5.80–16.00)						
	Chronological age	10.12 ± 2.24	10.29 (6.06–13.95)	1.41	1.80	−2.908	0.004	0.598 (0.545–0.646)	< 0.001
	ChatGPT 2nd estimation	9.92 ± 1.71	9.60 (6.20–15.20)						

Mean ± standard deviation (*SD*) and median (minimum–maximum) values are presented

*MAE* mean absolute error, *RMSE* root mean square error, *x* Wilcoxon signed-rank test, *y* intraclass correlation coefficient (*ICC*) with 95% confidence intervals (*CI*)

**Fig. 7:**
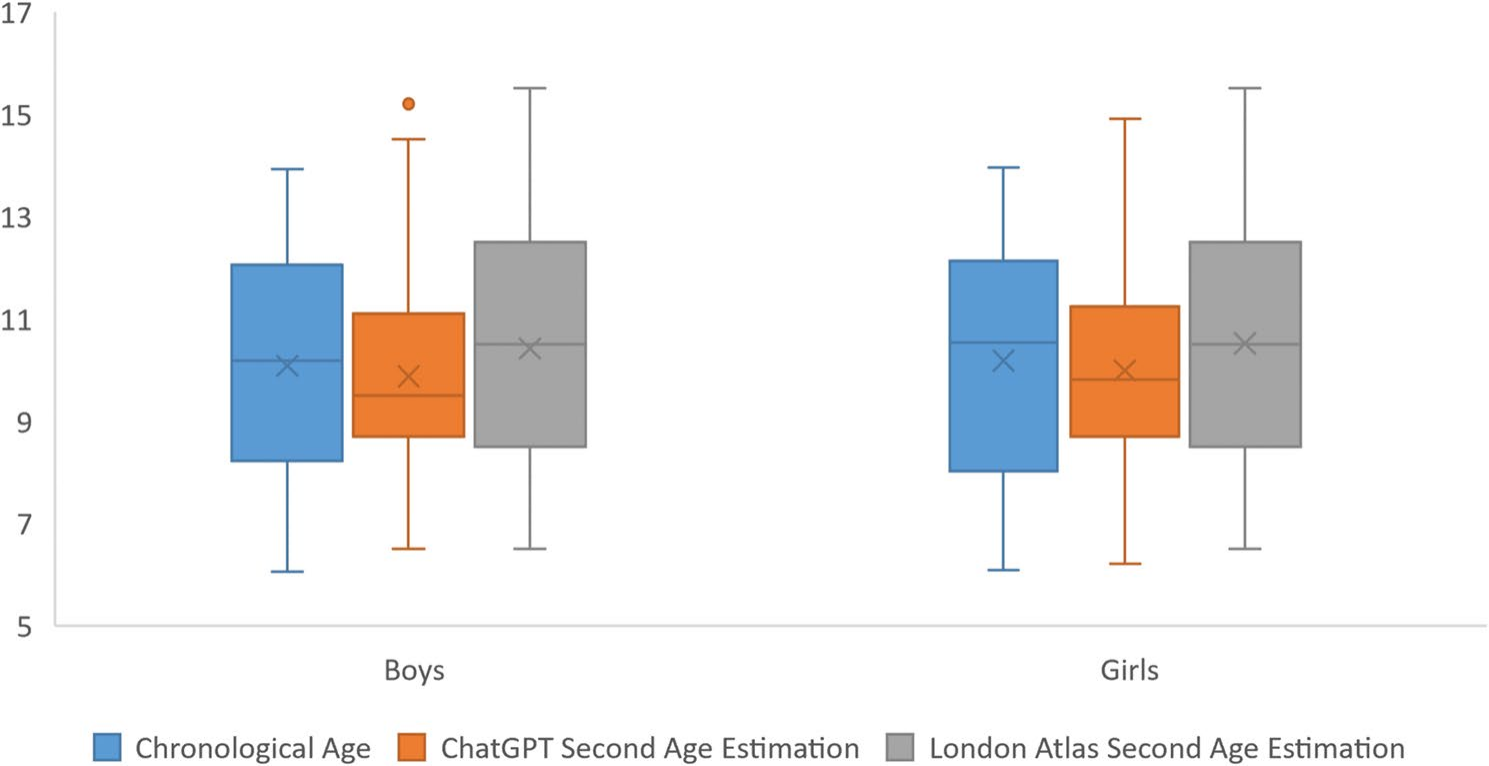
Comparison of chronological age in boys and girls with second age estimations by ChatGPT and London Atlas

In boys, no significant difference was observed between second-round ChatGPT estimations and CA (median: 9.5 years vs. 10.17 years, respectively; *p =*.054; ICC = 0.612). However, second-round predictions for girls (median: 9.8 years vs. 10.53 years; *p =*.034; ICC = 0.583) and for the overall sample (median: 9.6 years vs. 10.29 years; *p =*.004; ICC = 0.598) remained significantly lower (Table 5; Fig. 7).

The MAE and RMSE for ChatGPT were 1.47–1.37 and 1.86–1.77 years in boys, 1.47–1.45 and 1.82 years in girls, and 1.47–1.41 and 1.84–1.80 years overall for the first and second estimations, respectively (Table 5).

Bland–Altman analysis revealed that for the first round, the lower and upper LOAs between ChatGPT and CA were − 2.697 and + 4.082 years in boys, − 3.106 and + 3.864 years in girls, and − 2.913 and + 3.984 years overall. For the second round, the LOA were − 3.250 and + 3.660 years in boys, − 3.363 and + 3.753 years in girls, and − 3.304 and + 3.704 years overall (Table 3).

### London Atlas versus chronological age

Both rounds of LA estimations differed significantly from CA (*p <*.001). However, agreement with CA was better than for ChatGPT (boys: ICC = 0.902 and 0.901 in the first and second rounds, respectively; girls: ICC = 0.816 and 0.835, respectively; overall: ICC = 0.858 and 0.867, respectively; all *p <*.001) (Table 6; Figs. 6 and 7).

**Table 6:** Comparison of chronological age and London Atlas–based age estimation with error and reliability metrics

		Mean ± SD	Median (Min–Max)	MAE	RMSE	Test Statistic	pₓ Value	ICC (95% CI)	*p*_γ_ Value
Boys	Chronological age	10.07 ± 2.24	10.17 (6.06–13.92)	0.77	1.04	−5.146	< 0.001	0.902 (0.878–0.920)	< 0.001
	London Atlas 1 st estimation	10.34 ± 2.29	10.50 (6.50–15.50)						
	Chronological age	10.07 ± 2.24	10.17 (6.06–13.92)	0.79	1.06	−6.230	< 0.001	0.901 (0.877–0.920)	< 0.001
	London Atlas 2nd estimation	10.41 ± 2.29	10.50 (6.50–15.50)						
Girls	Chronological age	10.18 ± 2.24	10.53 (6.08–13.95)	0.95	1.41	−4.254	< 0.001	0.816 (0.775–0.850)	< 0.001
	London Atlas 1 st estimation	10.45 ± 2.32	10.50 (6.50–15.50)						
	Chronological age	10.18 ± 2.24	10.53 (6.08–13.95)	0.91	1.36	−5.352	< 0.001	0.835 (0.798–0.866)	< 0.001
	London Atlas 2nd estimation	10.51 ± 2.37	10.50 (6.50–15.50)						
Overall	Chronological age	10.12 ± 2.24	10.29 (6.06–13.95)	0.86	1.24	−6.632	< 0.001	0.858 (0.836–0.878)	< 0.001
	London Atlas 1 st estimation	10.40 ± 2.31	10.50 (6.50–15.50)						
	Chronological age	10.12 ± 2.24	10.29 (6.06–13.95)	0.85	1.22	−8.191	< 0.001	0.867 (0.846–0.885)	< 0.001
	London Atlas 2nd estimation	10.46 ± 2.33	10.50 (6.50–15.50)						

Mean ± standard deviation (SD) and median (minimum–maximum) values are presented

*MAE* mean absolute error, *RMSE* root mean square error, *x* Wilcoxon signed-rank test, *y* intraclass correlation coefficient (*ICC*) with 95% confidence intervals (*CI*)

The MAE and RMSE for the LA were 0.77–0.79 and 1.04–1.06 years in boys, 0.95–0.91 and 1.41–1.36 years in girls, and 0.86–0.85 and 1.24–1.22 years overall for the first and second estimations, respectively (Table 6).

Bland–Altman analysis demonstrated narrower limits of agreement for the LA. For the first round, the lower and upper LOA were − 2.234 and + 1.708 years in boys, − 2.994 and + 2.436 years in girls, and − 2.641 and + 2.099 years overall. For the second round, the LOA were − 2.312 and + 1.644 years in boys, − 2.929 and + 2.267 years in girls, and − 2.639 and + 1.975 years overall (Table 3).

### Age-group analysis

Comparison of ChatGPT estimations with CA by age group revealed significant differences across most age groups (Tables 7 and 8). In the first round, only the 8-year-old group among boys (*p =*.693) and the 8- (*p =*.107) and 9-year-old (*p =*.115) groups for girls showed no significant differences; all other groups differed significantly. In the second round, all age groups in boys differed significantly, and only the 9-year-old group among girls showed no significant difference (*p =*.742).

**Table 7:** Comparison of chronological age and ChatGPT first estimation by age group and sex with error and reliability metrics

		Chronological Age (Median or Mean ± SD)	ChatGPT 1 st Estimation (Median or Mean ± SD)	MAE	RMSE	Test Statistic	*p* Value
6.00–6.99	Boys	6.52 (6.06–6.92)	7.70 (6.30–12.10)	1.38	1.86	9.000	<.001^x^
	Girls	6.57 (6.08–6.92)	8.50 (5.80–11.00)	1.94	2.11	4.000	<.001^x^
7.00–7.99	Boys	7.63 (7.01–7.99)	8.05 (6.20–9.90)	0.77	0.94	173.000	.003^x^
	Girls	7.68 (7.01–7.98)	8.30 (6.50 − 10.70)	1.07	1.31	197.500	<.001^x^
8.00–8.99	Boys	8.47 ± 0.23	8.56 ± 1.22	0.91	1.19	−0.398	.693^y^
	Girls	8.35 (8.03–8.92)	9.00 (7.10–11.20)	0.90	1.16	142.500	.107^x^
9.00–9.99	Boys	9.47 (9.01–9.99)	8.70 (6.30–12.20)	1.24	1.49	726.000	<.001^x^
	Girls	9.61 (9.02–9.94)	8.70 (6.70–13.10)	1.24	1.48	329.000	.115^x^
10.00–10.99	Boys	10.36 (10.07–10.94)	9.20 (7.80–13.00)	1.26	1.48	665.500	<.001^x^
	Girls	10.61 (10.03–10.99)	9.90 (6.80–13.00)	1.13	1.42	874.000	.009^x^
11.00–11.99	Boys	11.43 (11.03–11.90)	9.80 (7.40–15.00)	1.79	2.07	817.000	<.001^x^
	Girls	11.43 (11.02–11.99)	10.70 (7.40–14.10)	1.49	1.81	685.000	.027^x^
12.00–12.99	Boys	12.62 (12.02–12.98)	10.95 (8.30–16.00)	1.85	2.18	916.000	<.001^x^
	Girls	12.51 ± 0.27	11.11 ± 1.33	1.55	1.92	6.708	<.001^y^
13.00–13.99	Boys	13.31 (13.02–13.92)	11.05 (8.20–15.00)	2.54	2.90	652.000	<.001^x^
	Girls	13.41 (13.03–13.95)	11.00 (6.60–13.10)	2.46	2.79	820.000	<.001^x^

Median (minimum–maximum) or mean ± standard deviation (SD) values are reported according to the distribution

*MAE* mean absolute error, *RMSE* root mean square error

ˣ: Wilcoxon signed-rank test; ʸ: paired *t*-test

**Table 8:** Comparison of chronological age and ChatGPT second estimation by age group and sex with error and reliability metrics

		Chronological Age (Median or Mean ± SD)	ChatGPT 2nd Estimation (Median or Mean ± SD)	MAE	RMSE	Test Statistic	*p* Value
6.00–6.99	Boys	6.52 (6.06–6.92)	8.50 (6.50–14.50)	2.20	2.68	0.000	<.001^x^
	Girls	6.53 ± 0.22	8.72 ± 1.41	2.20	2.56	−8.740	<.001^x^
7.00–7.99	Boys	7.63 (7.01–7.99)	8.55 (6.70–11.10)	1.05	1.32	37.000	<.001^x^
	Girls	7.68 (7.01–7.98)	8.70 (6.30–11.80)	1.45	1.76	107.500	<.001^x^
8.00–8.99	Boys	8.47 ± 0.23	8.98 ± 0.96	0.84	1.07	−3.018	.005^y^
	Girls	8.35 (8.03–8.92)	8.90 (6.40–10.80)	0.80	1.01	118.000	.032^x^
9.00–9.99	Boys	9.47 (9.01–9.99)	9.20 (7.00–11.60)	0.78	0.99	615.500	.041^x^
	Girls	9.61 (9.02–9.94)	9.50 (7.10–13.00)	1.06	1.38	216.000	.742^x^
10.00–10.99	Boys	10.36 (10.07–10.94)	9.60 (7.70–12.60)	1.17	1.38	550.500	.026^x^
	Girls	10.61 (10.03–10.99)	9.90 (6.90–14.20)	1.21	1.51	853.500	.007^x^
11.00–11.99	Boys	11.43 (11.03–11.90)	10.20 (7.20–15.20)	1.65	1.99	778.000	<.001^x^
	Girls	11.43 (11.02–11.99)	10.60 (8.00–13.60)	1.33	1.70	844.500	<.001^x^
12.00–12.99	Boys	12.62 (12.02–12.98)	11.50 (8.20–14.50)	1.54	1.92	856.000	<.001^x^
	Girls	12.52 (12.06–12.98)	11.15 (8.60–15.10)	1.66	1.91	737.000	<.001^y^
13.00–13.99	Boys	13.31 (13.02–13.92)	12.10 (8.90–13.80)	1.76	2.23	648.500	<.001^x^
	Girls	13.41 (13.03–13.95)	11.80 (6.70–13.80)	1.89	2.31	814.000	<.001^x^

Median (minimum–maximum) or mean ± standard deviation (SD) values are reported according to the distribution

*MAE* mean absolute error, *RMSE* root mean square error

ˣ: Wilcoxon signed-rank test; ʸ: paired *t*-test

LA estimations showed fewer significant differences relative to CA (Tables 9 and 10). In the first round, no significant differences were observed in the 9- (*p =*.279), 11- (*p =*.149), or 13-year-old (*p =*.475) groups among boys or in the 8- (*p =*.202), 11- (*p =*.086), 12- (*p =*.170), or 13-year-old (*p =*.064) groups among girls. In the second round, no significant differences were detected in the 11- (*p =*.053) or 13-year-old (*p =*.401) groups for boys or in the 7- (*p =*.459) or 13-year-old (*p =*.087) groups for girls.

**Table 9:** Comparison of chronological age and London Atlas first estimation by age group and sex with error metrics

		Chronological Age (Median. Min–Max)	London Atlas 1 st Estimation (Median. Min–Max)	MAE	RMSE	Test Statistic	*p* Value
6.00–6.99	Boys	6.52 (6.06–6.92)	6.50 (6.50–12.50)	0.56	1.21	159.000	0.031
	Girls	6.57 (6.08–6.92)	6.50 (6.50–13.50)	1.09	2.05	76.500	0.002
7.00–7.99	Boys	7.63 (7.01–7.99)	7.50 (6.50–9.50)	0.64	0.77	196.500	0.004
	Girls	7.68 (7.01–7.98)	7.50 (6.50–13.50)	1.05	1.45	369.000	0.040
8.00–8.99	Boys	8.41 (8.07–8.99)	9.50 (7.50–10.50)	0.97	1.08	74.000	< 0.001
	Girls	8.35 (8.03–8.92)	8.50 (7.50–10.50)	0.93	1.08	158.000	0.202
9.00–9.99	Boys	9.47 (9.01–9.99)	9.50 (7.50–11.50)	0.89	1.10	364.500	0.279
	Girls	9.61 (9.02–9.94)	9.50 (7.50–11.50)	0.67	0.85	105.500	0.005
10.00–10.99	Boys	10.36 (10.07–10.94)	10.50 (8.50–13.50)	0.84	1.06	216.500	0.016
	Girls	10.61 (10.03–10.99)	10.50 (6.50–13.50)	0.91	1.24	340.000	0.007
11.00–11.99	Boys	11.43 (11.03–11.90)	11.50 (6.50–13.50)	0.85	1.21	353.000	0.149
	Girls	11.43 (11.02–11.99)	11.50 (8.50–13.50)	1.14	1.32	347.500	0.086
12.00–12.99	Boys	12.62 (12.02–12.98)	13.00 (10.50–13.50)	0.70	0.80	301.500	0.024
	Girls	12.52 (12.06–12.98)	12.50 (10.50–13.50)	0.69	0.83	307.500	0.170
13.00–13.99	Boys	13.31 (13.02–13.92)	13.50 (10.50–15.50)	0.73	1.03	379.000	0.475
	Girls	13.41 (13.03–13.95)	13.50 (6.50–15.50)	1.03	1.98	548.500	0.064

Median (minimum–maximum) values are presented

*MAE* mean absolute error, *RMSE* root mean square error

*p*-values were obtained using the Wilcoxon signed-rank test

**Table 10:** Comparison of chronological age and London Atlas second estimation by age group and sex with error metrics

		Chronological Age (Median. Min–Max)	London Atlas 2nd Estimation (Median. Min–Max)	MAE	RMSE	Test Statistic	*p* Value
6.00–6.99	Boys	6.52 (6.06–6.92)	6.50 (6.50–12.50)	0.52	1.18	153.000	0.023
	Girls	6.57 (6.08–6.92)	6.50 (6.50–13.50)	0.93	1.96	65.000	0.001
7.00–7.99	Boys	7.63 (7.01–7.99)	7.50 (6.50–10.50)	0.74	1.01	238.500	0.022
	Girls	7.68 (7.01–7.98)	7.50 (6.50–13.50)	0.91	1.34	493.500	0.459
8.00–8.99	Boys	8.41 (8.07–8.99)	9.50 (7.50–10.50)	0.94	1.11	33.500	< 0.001
	Girls	8.35 (8.03–8.92)	9.50 (6.50–10.50)	0.95	1.12	101.000	0.011
9.00–9.99	Boys	9.47 (9.01–9.99)	10.00 (7.50–11.50)	0.91	1.08	231.000	0.006
	Girls	9.61 (9.02–9.94)	10.50 (8.50–12.50)	0.78	0.98	69.500	< 0.001
10.00–10.99	Boys	10.36 (10.07–10.94)	10.50 (8.50–12.50)	0.76	0.97	201.500	0.009
	Girls	10.61 (10.03–10.99)	10.50 (6.50–13.50)	1.02	1.32	318.000	0.003
11.00–11.99	Boys	11.43 (11.03–11.90)	11.50 (6.50–13.50)	0.98	1.32	312.500	0.053
	Girls	11.43 (11.02–11.99)	12.00 (9.50–14.50)	1.06	1.24	314.000	0.035
12.00–12.99	Boys	12.62 (12.02–12.98)	13.50 (10.50–15.50)	0.78	0.89	306.000	0.028
	Girls	12.52 (12.06–12.98)	12.50 (10.50–13.50)	0.62	0.76	210.500	0.008
13.00–13.99	Boys	13.31 (13.02–13.92)	13.50 (11.50–15.50)	0.61	0.86	387.000	0.401
	Girls	13.41 (13.03–13.95)	13.50 (6.50–15.50)	0.92	1.84	538.000	0.087

Median (minimum–maximum) values are presented

*MAE* mean absolute error, *RMSE* root mean square error

*p*-values were obtained using the Wilcoxon signed-rank test

Error magnitude varied non-linearly by age. For ChatGPT, MAE values were highest in the 6-, 12-, and 13-year-old groups and lowest in the 8-year-old group. For example, in Round 1, MAE ranged from 1.38 to 2.54 years in boys and 1.94–2.46 years in girls, and in Round 2 from 0.78 to 2.20 years in boys and 0.80–2.20 years in girls (Tables 7 and 8). By contrast, the LA exhibited lower and comparatively stable errors across age categories, with MAE generally ranging from 0.52 to 1.14 in boys and 0.62–1.14 in girls (Tables 9 and 10), accompanied by narrower RMSE ranges than ChatGPT.

## Discussion

This study compared the accuracy and reproducibility of age estimations derived from PRs using ChatGPT and the LA method. ChatGPT estimations showed statistically significant differences from CA in multiple age groups, whereas the LA yielded fewer discrepancies. Repeated estimations revealed moderate consistency for ChatGPT (ICC = 0.703) and near-perfect agreement for the LA (ICC = 0.960). These findings indicate that AI-based approaches such as ChatGPT hold potential for dental age estimation but, at present, remain less reliable than established methods.

In addition to this moderate agreement, the present study revealed that ChatGPT produced inconsistent results when identical PRs were re-evaluated. Such inconsistency underscores the model’s instability in repeated diagnostic tasks, which is a more critical limitation than the observed overestimation or underestimation tendencies. Given that reproducibility is essential in forensic and clinical dental applications, these findings highlight the need for cautious interpretation of ChatGPT-based age estimations until model refinement ensures stable and repeatable outputs.

Previous studies have frequently emphasized the influence of population differences on the accuracy of dental age estimation methods. Sezer et al. compared the LA, Haavikko, and Cameriere methods in Turkish children and report performance variations related to population characteristics [8]. Similarly, Ozveren et al. uncovered systematic over- and underestimation tendencies with the Willems and Cameriere methods in the Turkish population, noting critical implications for forensic practice [9]. Kış et al. observed systematic overestimation with the Willems method and underestimation in certain age groups using the Cameriere method, recommending population-specific regression formulae for improved accuracy [18]. These findings are consistent with our observation that ChatGPT tended to overestimate age in younger groups and underestimate age in older groups, suggesting that population-specific calibration and model training could enhance accuracy in AI-based approaches.

The accuracy of the LA and its potential susceptibility to sex differences have also been previously reported. Koç et al. found that the LA performed particularly well in individuals aged 12 years and older but exhibited systematic deviations in younger children [7]. Our findings, in which the LA demonstrated fewer age-group discrepancies and higher reproducibility than ChatGPT, are consistent with those observations.

AI-based approaches in dental age estimation have recently attracted growing interest. Matthijs et al. developed a CNN-based system for automated classification of mandibular tooth development stages from PRs, highlighting its potential to reduce observer dependency and improve standardization [19]. Ong et al. proposed a deep learning model based on the Demirjian method, achieving high accuracy but reporting poorer performance in incisors among younger age groups, paralleling our observation that ChatGPT tended to overestimate age in some younger children [20]. Kokomoto et al. developed a two-stage deep learning model for fully automated dental age estimation and report a mean absolute error of only 0.261 years, indicating clinically acceptable performance and demonstrating the potential of AI for time-efficient, standardized analysis [15]. Unlike these studies, the present research evaluated the LLM ChatGPT for direct age estimation, representing one of the first systematic assessments of LLM-based image interpretation in this context and contributing novel insights to the literature.

Aşar et al. evaluated GPT-4–based LLMs for detecting supernumerary teeth on periapical radiographs and demonstrated superior performance with a customized model, achieving 91% accuracy [16]. This supports the notion that task-specific AI approaches can enhance performance and suggests that ChatGPT may achieve greater accuracy in age estimation when trained specifically for this task. Similarly, Yilmaz et al. developed a deep learning model capable of differentiating the critical forensic age threshold of 12 years from PRs with 94% accuracy, underscoring the potential of AI in forensic age assessment [21]. Our finding that ChatGPT can directly predict CA from PRs, similar to classification-based approaches, highlights its promise for forensic applications, although ethical considerations and fairness issues remain crucial.

Population-specific reference data have been shown to improve the accuracy of age estimation as demonstrated by Vidisdottir et al., who created a dental development database tailored to the Icelandic population [22]. In our study, deviations observed with the use of the LA in certain age groups and ChatGPT’s age-dependent bias (overestimating in younger and underestimating in older children) further support the influence of population differences on both traditional and AI-based methods. Moreover, sex-related differences and the established importance of population-specific reference standards suggest that AI models such as ChatGPT may benefit from population-specific training to achieve optimal performance.

Silva et al. evaluated ChatGPT-3.5 in identifying radiolucent lesions on PRs and found limited accuracy in image-based diagnostic tasks, aligning with our finding that ChatGPT is not yet fully optimized for complex visual decision-making processes, such as age estimation [23]. Model optimization and task-specific fine-tuning may therefore improve its performance.

Dental age estimation plays a critical role in both forensic medicine and clinical disciplines such as orthodontics, particularly when legal age thresholds, such as those for criminal responsibility, are involved [9]. The tendency of ChatGPT to overestimate younger children’s ages and underestimate older children’s ages is therefore a noteworthy finding with potential forensic implications. While our results demonstrate that ChatGPT has potential for complex decision-making tasks, its current accuracy and reproducibility remain inferior to those of traditional methods such as the LA.

This study has limitations. First, all analyses were based solely on children from Turkey’s Thrace region, and different outcomes may be observed in other geographic or ethnic populations. Second, we used a general-purpose ChatGPT model rather than a task-specific or customized version as employed by Aşar et al. [16], which may have influenced performance.

From a methodological perspective, ChatGPT inherently operates with stochastic sampling, meaning that slight variations in output may occur even under identical input conditions. This non-deterministic behavior represents another limitation that future research should address by using API-based configurations with fixed parameters (e.g., temperature and seed values) to ensure deterministic and reproducible output generation.

Although only fully anonymized radiographs were used, the use of public AI interfaces such as ChatGPT raises broader concerns regarding data security and compliance with healthcare privacy standards (e.g., Health Insurance Portability and Accountability Act (HIPAA), General Data Protection Regulation (GDPR), and local equivalents). Future implementations should prioritize secure, institutionally hosted, or API-based environments to ensure complete regulatory compliance [24].

Future research should focus on developing customized ChatGPT models trained on population-specific datasets and conducting multicenter studies to further explore the integration of AI into forensic and clinical dental applications.

## Conclusions

This study demonstrates that the multimodal LLM ChatGPT can be employed for direct dental age estimation from PRs. Although ChatGPT shows promise for complex decision-making tasks such as age estimation, its accuracy and consistency remain lower than those of established methods such as the LA. The latter exhibited greater reproducibility and fewer systematic errors, whereas ChatGPT tended to overestimate age in younger children and underestimate age in older ones.

Moreover, the inconsistent predictions produced by ChatGPT across repeated evaluations highlight a critical limitation regarding its reliability. Such variability indicates that, beyond simple over- or underestimation tendencies, the model’s current output stability is insufficient for dependable clinical or forensic use. Therefore, its results should be interpreted with caution until future versions achieve reproducible predictions for identical inputs.

Nevertheless, this study highlights the growing potential of LLMs in forensic and clinical dentistry. With population-specific training and task-focused optimization, ChatGPT and similar AI-based approaches may achieve accuracy comparable to or even exceeding that of traditional methods. Future research should prioritize customized model development, multicenter validation across diverse populations, and integration of these technologies into forensic and clinical workflows.

## Data Availability

The datasets generated and analyzed during the current study are not publicly available due to institutional data protection policies and ethical restrictions but are available from the corresponding author on reasonable request.

## Abbreviations

LLM: Large Language Model
PR: Panoramic Radiograph
LA: London Atlas
CA: Chronological Age
MAE: Mean Absolute Error
RMSE: Root Mean Squared Error
ICC: Intraclass Correlation Coefficient
AI: Artificial Intelligence
CNN: Convolutional Neural Network
API: Application Programming Interface
SD: Standard Deviation
CI: Confidence Intervals
LOA: Limit of Agreement
